# Modulation of Neural Spiking in Motor Cortex–Cerebellar Networks during Sleep Spindles

**DOI:** 10.1523/ENEURO.0150-23.2024

**Published:** 2024-05-02

**Authors:** Pierson Fleischer, Aamir Abbasi, Tanuj Gulati

**Affiliations:** ^1^Department of Biomedical Sciences, Center for Neural Science and Medicine, Cedars–Sinai Medical Center, Los Angeles, California 90048; ^2^Department of Neurology, Cedars–Sinai Medical Center, Los Angeles, California 90048; ^3^Department of Medicine, David Geffen School of Medicine; and Department of Bioengineering, Henry Samueli School of Engineering, University of California–Los Angeles, Los Angeles, California 90095

**Keywords:** cerebellum, motor cortex, sleep

## Abstract

Sleep spindles appear to play an important role in learning new motor skills. Motor skill learning engages several brain regions with two important areas being the motor cortex (M1) and the cerebellum (CB). However, the neurophysiological processes in these areas during sleep, especially how spindle oscillations affect local and cross-region spiking, are not fully understood. We recorded an activity from the M1 and cerebellar cortex in eight rats during spontaneous activity to investigate how sleep spindles in these regions are related to local spiking as well as cross-region spiking. We found that M1 firing was significantly changed during both M1 and CB spindles, and this spiking occurred at a preferred phase of the spindle. On average, M1 and CB neurons showed most spiking at the M1 or CB spindle peaks. These neurons also developed a preferential phase locking to local or cross-area spindles with the greatest phase-locking value at spindle peaks; however, this preferential phase locking was not significant for cerebellar neurons when compared with CB spindles. Additionally, we found that the percentage of task-modulated cells in the M1 and CB that fired with nonuniform spike phase distribution during M1/CB spindle peaks were greater in the rats that learned a reach-to-grasp motor task robustly. Finally, we found that spindle band LFP coherence (for M1 and CB LFPs) showed a positive correlation with success rate in the motor task. These findings support the idea that sleep spindles in both the M1 and CB recruit neurons that participate in the awake task to support motor memory consolidation.

## Significance Statement

Neural processing during sleep spindles is linked to memory consolidation. However, little is known about sleep activity in the cerebellum (CB) and whether CB spindles can affect spiking activity in local or distant areas. We report the effect of sleep spindles on neuron activity in the M1 and CB—specifically their firing rate and phase locking to spindle oscillations. Our results indicate that awake practice neuronal activity is tempered during local M1 and CB spindles, and during cross-region spindles, which may support motor skill learning. We describe spiking dynamics in motor network spindle oscillations that may aid in the learning of skills. Our results support the sleep reactivation hypothesis and suggest that awake M1 activity may be reactivated during CB spindles.

## Introduction

Sleep-related neural processing is required for the consolidation of new motor skills (Rasch and Born, 2013; Gulati et al., 2014, 2017; Ramanathan et al., 2015; Miyamoto et al., 2016; Latchoumane et al., 2017; Kim et al., 2019). Sleep spindles, which are 10–16 Hz bursts of activity as detected in EEG signals and local field potentials (LFPs), are postulated to have a chief role in off-line processing in a variety of studies that span declarative memory tasks (Gais et al., 2002; Clemens et al., 2005, 2006) to motor learning paradigms (Walker et al., 2002; Fogel and Smith, 2006; Nishida and Walker, 2007; Barakat et al., 2011; Johnson et al., 2012; Ramanathan et al., 2015). Conventionally, neocortical sleep spindles are believed to have a thalamocortical origin (Steriade et al., 1993; Rasch and Born, 2013); however, recent studies have indicated cerebellar involvement (Xu et al., 2021, 2022). Our recent work has shown that the motor cortex (M1) and cerebellum (CB) develop an awake low-frequency coherence [low-frequency oscillatory (LFO) activity, 1–4 Hz] as rats learn a skilled reaching task (Fleischer et al., 2023). An intriguing possibility is that neural activity patterns in these two structures during “off-line” periods, or time away from training (such as sleep), contribute to corticocerebellar plasticity during skill learning. This possibility gains more credibility with the evidence that “reactivation” of awake training activity patterns during sleep promotes motor skill learning (Gulati et al., 2014; Yang et al., 2014; Ramanathan et al., 2015; Cousins et al., 2016; Kim et al., 2019). Moreover, sleep-dependent consolidation of motor skills engages both the M1 and CB (Doyon and Benali, 2005; Canto et al., 2017; Doyon et al., 2018). However, the specific neuronal activity patterns in these areas during local and cross-area spindle-mediated interactions are not fully understood. Despite the extensive work that links sleep spindles to memory consolidation, relatively little is understood about the relationship between these oscillations and spiking activity in the larger motor network.

Temporally precise neural spiking is at the core of regulating changes in synaptic efficacy (Hebb, 1949; Bi and Poo, 2001). Phase-locked spiking of prefrontal cortex and M1 neurons to sleep spindles has been shown in prior work (Peyrache et al., 2011; Gardner et al., 2013; Sela et al., 2016; Silversmith et al., 2020). However, it is unknown what the spiking dynamics are around M1 spindles in a subcortical structure like the CB or vice versa—what happens to neocortical spiking during CB spindles. This is a crucial question as the M1 and the CB are densely, reciprocally connected (Kelly and Strick, 2003; Guo et al., 2021), and neocortical spindles have a cerebellar origin (Xu et al., 2021).

To this end, we simultaneously recorded LFPs and spiking activity from the M1 and cerebellar cortex of sleeping rats and examined the spike timing relative to ongoing spindles in both regions. We parsed spindles into their component cycles, which allowed us to analyze the spiking activity in detail during the evolution of spindles. This analysis revealed that M1 and cerebellar neurons significantly increase their spike rates during M1 and CB spindle peaks. M1 neurons also experienced significantly increased spindle-peak phase locking to M1 and CB spindles as compared with spindle tails. CB neurons experienced significantly increased phase locking to M1 spindles; however, this phenomenon was not seen for CB neurons with CB spindles. Additionally, we also observed a significantly greater number of M1 and CB neurons showing significantly nonuniform spike phase distribution during M1 and CB spindles in the rats that gained expertise in the dexterous reach-to-grasp motor skill versus the ones that did not. Finally, we also found that spindle band LFP coherence magnitude in M1 and CB LFPs was positively correlated with awake motor task success rate. Our work here expands our understanding of how spiking activity is changed during sleep spindles in the larger motor network that may be linked to skill learning.

## Materials and Methods

### Animal model and surgical procedures

All procedures were conducted in accordance with protocols approved by the Institutional Animal Care and Use Committee at the Cedars-Sinai Medical Center. Adult male Long–Evans rats (*n* = 8; weight, 250–400 g; Charles River Laboratories) were housed in a 14 h/10 h light/dark cycle. All experiments were performed during the light cycle. No statistical methods were used to predetermine cohort size, but our sample sizes are similar to those reported in previous publications (Kargo and Nitz, 2004; Ramanathan et al., 2015, 2018; Sauerbrei et al., 2015; Gulati et al., 2017; Lemke et al., 2019; Fleischer et al., 2023). Animals were pair-housed before electrode implantation and then single-housed after to prevent damage to implants or to implement food restriction.

All surgical procedures were performed using sterile techniques under 1–4% isoflurane. Surgery involved cleaning and exposure of the skull and preparation of the skull surface using adhesive cement (C & B Metabond, Parkell) followed by implantation of the skull screws for referencing and overall head-stage stability. The analgesic regimen included the administration of 0.1 mg/kg body weight buprenorphine and 5 mg/kg body weight carprofen. Neural implanted rats were also administered 2 mg/kg body weight dexamethasone and 33 mg/kg body weight Sulfatrim for 5 d. Postsurgery, animals were allowed to recover for 5 d before further behavioral training and sleep recordings. Ground and reference screws were implanted posterior to lambda, contralateral to the recorded CB and contralateral to the neural recordings. For M1 recordings, 32-channel arrays (33 μm polyamide-coated tungsten microwire arrays) were lowered to a depth of 1,200–1,500 μm in either the left or right M1 depending on handedness. These were implanted centered at 0.5 mm anterior and 3 mm lateral to the bregma (Ramanathan et al., 2015; Lemke et al., 2019; Abbasi et al., 2021, 2024; Fleischer et al., 2023). For cerebellar recordings, we used 32–64-channel tetrodes (NeuroNexus) or shuttle-mounted polytrodes (Cambridge NeuroTech). The probes were lowered into the cerebellar cortex through a craniotomy centered at 12.5 mm posterior and 2.5–3 mm lateral to the bregma. Shuttle-mounted probes were moved across days and recorded from depths of 1.5–4 mm. Our target regions were the Simplex/Crus I and Crus II areas of the CB. Activity in these areas has shown modulation during upper limb motor behaviors (including our own recently published work) and in response to corticofugal fiber and forelimb stimulation (Atkins and Apps, 1997; Baker et al., 2001; Heck et al., 2007; Fleischer et al., 2023).

### Experimental design

Rats were acclimated to the behavioral box for at least 2 d and then exposed to a reach-to-grasp task for 5–10 trials to establish hand preference before neural probe implantation. Probe implantation was performed in the contralateral M1 and ipsilateral CB to the preferred hand. Thereafter, postrecovery rats underwent motor training and sleep recordings. Motor training involved training them on a reach-to-grasp motor task (Fleischer et al., 2023). During behavioral assessments, we monitored the animals and ensured that their body weights did not drop to <90% of their initial weight. We used an automated reach box for motor training, controlled by custom MATLAB scripts and an Arduino board. This setup requires minimal user intervention, as described previously (Wong et al., 2015). Each trial consisted of a pellet dispensed on the pellet tray, followed by an alerting beep indicating that the trial was beginning. They then had 15 s to reach their arms through the slot to grasp and retrieve the pellet. A real-time “pellet detector” using an infrared sensor centered over the pellet was used to determine when the pellet was moved, which indicated that the trial was over, and the door was closed. The rats underwent sleep recordings in the same box. For the sleep sessions, the pellet presentations stopped, and a spontaneous recording period ensued during which we analyzed the sleep. Electrophysiology recordings were taken throughout the full extent of the behavioral training and sleep, which consisted of one to two reach sessions of 60–100 trials/d for 5 d, and sleep sessions of 1–2 h.

### In vivo electrophysiology

Units and LFP activity were recorded using a 128-channel TDT-RZ2 system (Tucker-Davis Technologies). Spike data were sampled at 24,414 Hz, and LFP data were sampled at 1,017.3 Hz. ZIF (zero insertion force) clip-based digital head stages from Tucker-Davis Technologies were used that interface the ZIF connector and the Intan RHD2000 chip that uses 192× gain. We performed off-line spike sorting on the recorded spike data using Plexon (where spike times and waveform snippets were saved) or Spyking Circus (Yger et al., 2018; where spike data were saved at 24,414 Hz). The average firing rate of M1 neurons was 1.71 ± 0.03 Hz and Cb units 6.48 ± 0.34 Hz.

### Behavioral analysis

Behavioral analysis was performed using video recorded during experimental sessions. Reach videos were viewed and manually scored to obtain trial success. To characterize motor performance, we quantified pellet retrieval success rate (percentage of pellets successfully retrieved into the box). We classified animals as expert and nonexpert based on a success rate of at least 30% by Day 5. We used similar classification in our recent work, where we found that emergent low-frequency activity across corticocerebellar networks was restricted to animals that gained expertise in the task (Fleischer et al., 2023). Based on this classification, we found that four animals were experts and four animals did not achieve expertise in the task in 5 d of practice.

### Sleep classification

Sleep was detected through M1 LFP recordings. Each LFP channel in M1 was segmented into nonoverlapping 6 s windows. In each window, the power spectral density (PSD) was computed and averaged over the delta/SO (0.1–4 Hz) and gamma (30–60 Hz) frequency bands (Kim et al., 2019; Silversmith et al., 2020). Then a *k*-means classifier was used to cluster epochs into two clusters, non-rapid eye movement (NREM) sleep and rapid eye movement sleep (REM) /awake. Only long (>30 s, five consecutive windows) epochs of sleep were analyzed. Further analyses of both recorded areas used only the periods classified as non-REM sleep by this method.

### Spindle detection

**Figure 1.**
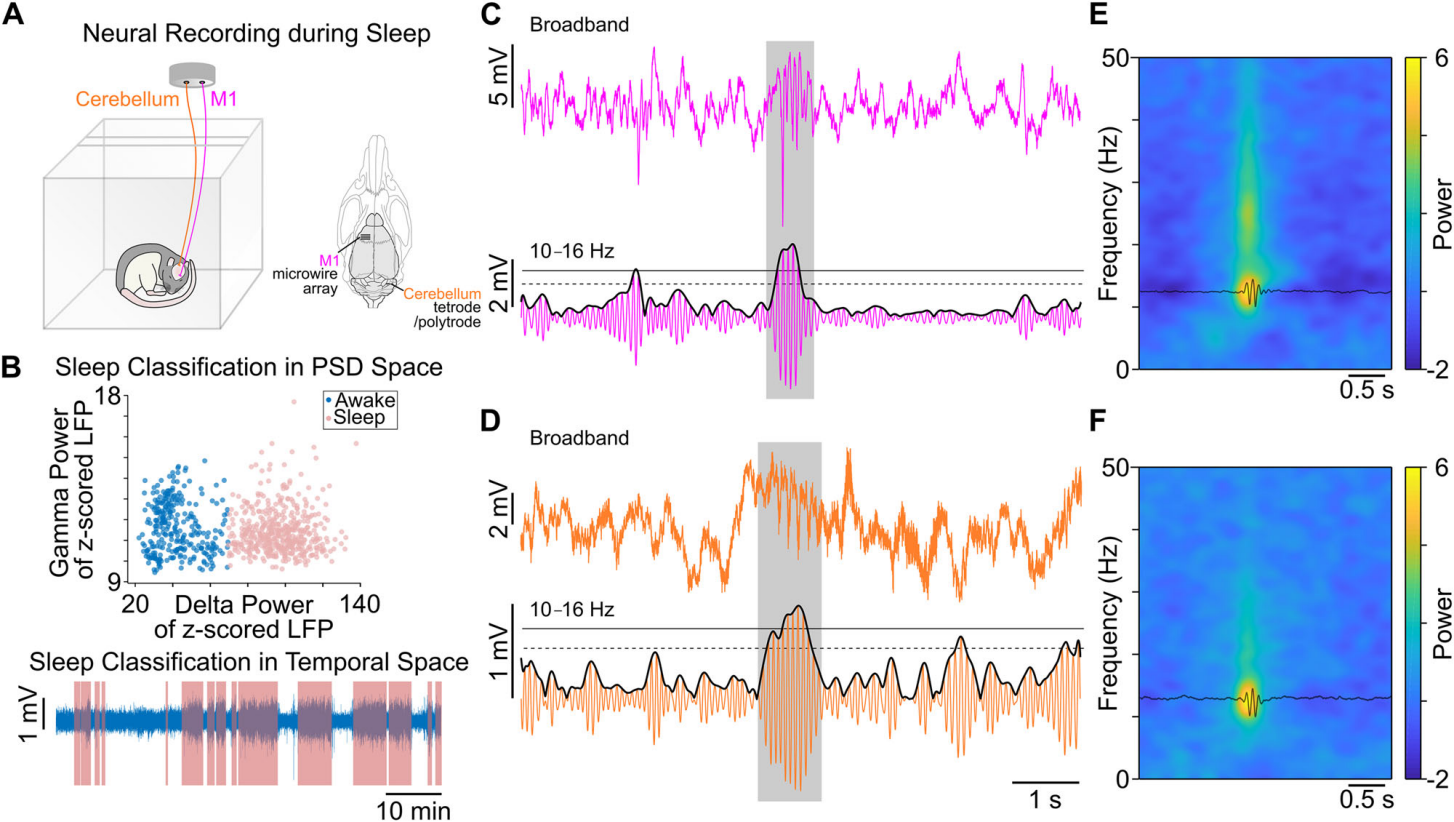
Recording setup and detecting NREM sleep. ***A***, Sleeping rat along with location of recording locations in the M1 and CB. ***B***, Example of sleep classification in both PSD (top) and temporal spaces. Each dot represents the PSD in *γ* (30–60 Hz) and *δ* (0.1–4 Hz) frequency bands during 6 s windows. Epochs were classified into two clusters using *k*-means clustering: awake/REM (blue) and NREM (red). The average LFP trace is plotted for an example sleep block at the bottom. ***C***, Example of a detected M1 spindle, highlighting the automatic method used for identification. The broadband LFP (top) is filtered in the spindle band and the spindle band components (bottom) are extracted. The spindle band envelope (black line; bottom) must have exceeded an upper threshold (solid black line; bottom) for one sample and a lower threshold (dashed black line; bottom) for at least 500 ms. Transparent shaded gray box represents a detected spindle. ***D***, Same as ***C*** showing cerebellar spindle detection. ***E***, Average M1 spindle-triggered waveform (black line) and spectrogram (heat map). ***F***, Average CB spindle-triggered waveform (black line) and spectrogram (heat map).

The spindle detection applied here is an algorithm that has been used recently (Sela et al., 2016; Kim et al., 2019; Silversmith et al., 2020; Simpson et al., 2023) and was applied separately to M1 and CB LFPs. Channels without obvious artifacts were first *z*-scored and averaged to form a virtual LFP channel. This signal was filtered in the spindle band (10–16 Hz) using a zero-phase shifted, third-order Butterworth filter. A smoothed envelope was calculated by computing the magnitude of the Hilbert transform of this signal and then convolving it with a Gaussian window (*α* = 2.5). Next, we determined two thresholds for spindle detection (Kim et al., 2019; Silversmith et al., 2020) based on the mean and standard deviation of the spindle band envelope during NREM sleep (lower, 1.5 SD; upper, 2.5 SD). Epochs during non-REM sleep in which the spindle envelope exceeded the upper threshold for at least one sample and the spindle power exceeded the lower threshold for at least 500 ms were considered spindles (Fig. 1*C*). Finally, spindles that were sufficiently close in time (<300 ms) were combined. For each spindle epoch, the peak of the spindle band LFP was identified. Spindles were aligned to this peak for generating average spindle waveforms and spike raster plots.

### Control spindle epochs

There is a certain degree of spiking, phase locking, and synchrony between neurons, which is expected even if neurons are not modulated by spindles. To account for such effects, we generated a control spindle distribution that had similar statistics to the true spindle epoch distribution. For each spindle epoch, two offsets were computed 5 and 10 s before the true spindle peak. The nearest maxima in the spindle band LFP to those offsets were taken as the spindle centers (also called spindle “peaks” henceforth) of the control epoch. Each analysis was jointly computed for the true spindle epochs (blue/teal) and the control spindle epochs (gray).

### Spike phase extraction

The following methods for spike phase extraction were used to assess the spiking structure within a spindle cycle and across spindles. We performed this analysis only for reach-modulated neurons during awake trainings. This was defined as ±1.97 SD task-related (reach-related) modulation over baseline. For both analyses, we first computed the same virtual signal used in spindle detection and then filtered the data in the spindle band (10–16 Hz). Next, we applied the Hilbert transform and took the angle at each sample to get a continuous representation of the relative spindle phase. For each spindle epoch and each neuron, the corresponding phase was collected at each spike event.

### Phase-locking value (PLV) and preferred spindle phase

We calculated the PLV as follows in order to assess the degree of phase consistency of spiking within spindle cycles. For a given neuron, across all spindle epochs, the phase of the spindle band LFP signal was collected at each detected action potential within a given cycle yielding a distribution of spike phases. Each phase value in this distribution was treated as a vector of magnitude one and angle equal to the phase:

$$\mathrm{Average}\;\mathrm{Spike}\;\mathrm{Phase}\;\;\mathrm{Vector}=\;\frac{1}{n}\;\overset{n}{\underset{i=0}{\sum }}\;e^{\theta_{i}}.$$

The average phase vector was computed according to the above equation (Fig. 3, green arrow). From this vector, we attained the PLV (vector magnitude) and the preferred spindle phase (vector angle). We calculated these measures for each neuron and each spindle cycle using the functions circ_r() and circ_mean(), respectively (from MATLAB's circular statistics toolbox). Linear mixed-effects models were used to test the significance of changes in the firing rate and PLV. Watson–Williams test of equal means was used to test the significance of differences in the preferred phase.

### Nonuniformity *z*-statistic and CDF

The PLV is closely related to Raleigh's *z-*statistic for circular nonuniformity. *z-*statistic is simply calculated as *z* = *n*(PLV)^2^. These *z-*statistics were then used to calculate the percentage of significantly nonuniform distributions across unit–LFP pairs with a significance threshold of *p* = 0.05. A significantly nonuniform distribution signifies phase preference for spikes of a unit to the spindle band of the LFP signal. For this analysis, we also compared the *z-*statistic of spiking over peak cycles of the cohort of experts to that of the nonexperts.

### Spindle band M1 and CB LFP coherence analysis

We measured LFP coherence during NREM sleep across all M1 and CB electrode pairs. First, we subtracted the common-mode referencing signal from M1 and CB LFPs using the median LFP signal from each region. At every timepoint, the median of all the LFP in M1 and CB was calculated and subtracted from every electrode in that region, respectively, in order to reduce common noise and volume conduction. We then computed LFP coherence between pairs of M1 and CB LFP channels during NREM sleep in nonoverlapping 10 s windows using the cohgramc function of the Chronux toolbox in MATLAB. By doing so, we got coherograms for every pair of M1 and CB electrode. We computed the spindle band coherence by taking the average of coherence in 10–16 Hz frequency range.

### Data availability

The datasets generated and analyzed in the current study are available from the corresponding author on reasonable request.

## Results

### Neural oscillation detection

We recorded extracellular LFP and spiking activity from the M1 and CB in eight rats that were trained on the reaching task interspersed with sleep for 5 d. Out of these eight rats, the four that achieved over 30% accuracy by Day 5 of training were classified as experts (D5 success rate, 49.46 ± 5.8%; mean ± SEM), and the other four were classified as nonexperts as their final success rate was under 30% on Day 5 (D5, 17.59 ± 2.33%; see Materials and Methods). Nonexpert animals were excluded from all the analyses presented here unless expressly stated otherwise. During “sleep blocks,” the animals were given the opportunity to sleep for ∼2 h. On average, NREM sleep was analyzed for 77.03 ± 5.4 min per day for expert animals and 100.76 ± 5.3 min per day for nonexpert animals (as detected through M1 LFPs; Fig. 1*B*; see Materials and Methods). During NREM sleep, we identified ongoing spindles in the M1 (∼3,389 per expert and ∼4,561 per nonexpert across 5 d) and CB (∼2,512 per expert and ∼4,860 per nonexpert across 5 d) using standard algorithms for automatic detection (Sela et al., 2016; Kim et al., 2019; Silversmith et al., 2020). Briefly, LFP channels were *z*-scored to standardize activity levels. The averaged signal was filtered in the spindle band (10–16 Hz). Periods in which spindle power exceeded an upper threshold for at least one sample or data point and a lower threshold for at least 500 ms were identified as spindles (see Materials and Methods; see Fig. 1*C,D* for M1 and cerebellar spindle example, respectively). We also looked at the spindle-triggered spectrogram of LFP in the M1 (Fig. 1*E*) and CB (Fig. 1*F*) which revealed an increase in power in the spindle-specific frequency band.

### Spindles properties in the M1 and CB

**Figure 2.**
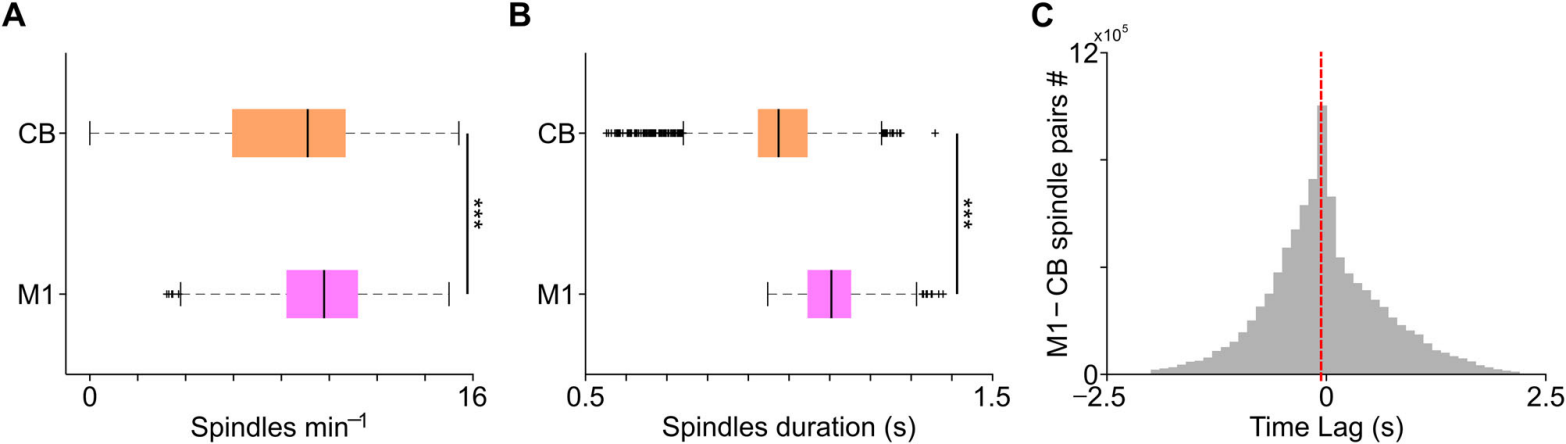
Spindle features in the M1 and CB. ***A***, Spindle rate in the M1 and CB depicted as a boxplot from all animals (*n* = 8). Lower and upper box boundaries are 25th and 75th percentiles, respectively; line inside the box is the median; lower and upper error lines are 10th and 90th percentiles, respectively; “+” indicates outliers outside these bounds. ***B***, Spindle duration in the M1 and CB from all animals (*n* = 8). ***C***, Distribution of time lag between M1 and CB spindles from all animals (*n* = 8). Vertical red line showing median lag between M1 and CB spindles. ***p < 0.001.

We looked at the features of spindles in the M1 and CB in terms of rate and duration. We found that the average rate of spindles in the M1 was significantly higher than the CB [Fig. 2*A*; M1, 9.58 ± 0.05 spindles min^−1^; CB, 8.16 ± 0.06 spindles min^−1^; mixed-effects model, *t*_(4,648)_ = −14.69; *p* = 7.822 × 10^−48^]. We also found that the spindle duration in the M1 was significantly longer than the CB [Fig. 2*B*; M1, 1.10 ± 0.001 s; CB, 0.97 ± 0.002 s; mixed-effects model, *t*_(4,648)_ = −47.13; *p* = 0). We also characterized the temporal relationship between the M1 and cerebellar spindles by looking at the time lag distribution of the spindle onset (Fig. 2*C*) which showed that there was a preponderance of cerebellar spindles leading M1 spindles with the median lag of −0.06 s.

### M1 and cerebellar spindle-associated spiking modulated by motor skill learning

**Figure 3.**
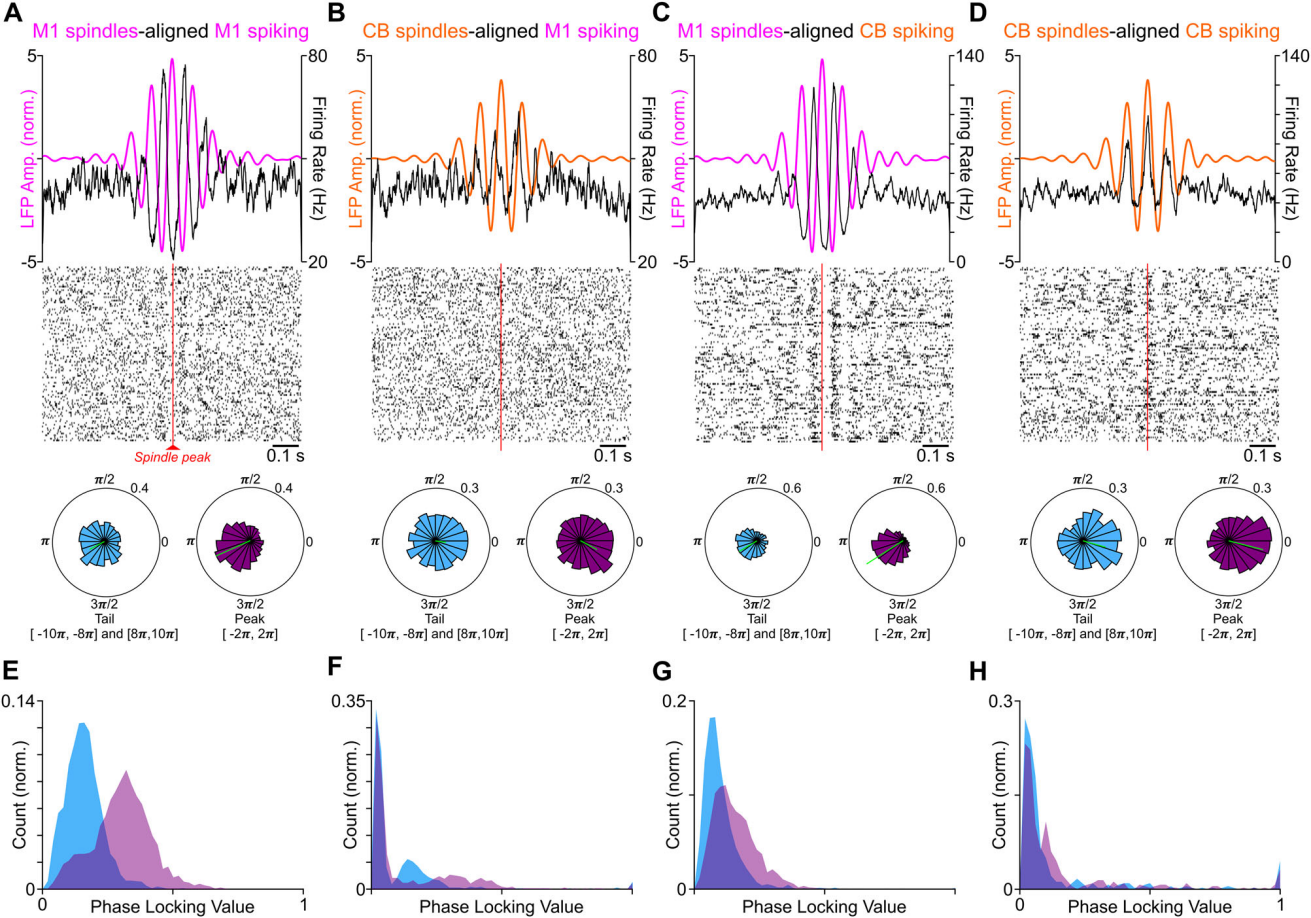
Spindle modulation of M1 and CB spiking. ***A***, M1 spindle-modulated spiking for an example M1 unit. The average M1 spindle band waveform (pink) is depicted on top along with the averaged firing rate of the M1 unit aligned to the spindle peak (black). Rasters of spike times are displayed below. Radial histograms at the bottom show the spike phase distributions of the same M1 unit at aligned spindle peaks (purple, two cycles around peak [−2π, 2π]) and tails (blue, [−10*π*, −8*π*] and [8*π*, 10*π*]). The average phase vector is overlayed in green. The magnitude and direction of this vector are defined as the PLV and preferred spindle phase, respectively. This was collected for all units. ***B***, Same as ***A*** but for the same M1 unit and its modulation around CB spindle. Conventions are the same. ***C***, Same as ***A*** but for CB unit and its modulation around M1 spindle. Conventions are the same. ***D***, Same as ***A*** but for the same CB unit and its modulation around CB spindle. Conventions are the same. ***E***, Distribution of PLV for all M1 unit–M1 spindle pairs around the spindle peak (purple) and tail (blue) from expert animals (*n* = 4). ***F***, Same as ***E*** but for M1 unit–CB spindle pairs. ***G***, Same as ***E*** but for CB unit–M1 spindle pairs. ***H***, Same as ***E*** but for CB unit–CB spindles pairs.

Recent work has shown that sleep spindles modulate awake practice/training activity in the healthy M1 as well as during stroke recovery (Kim et al., 2019, 2022; Silversmith et al., 2020; Lemke et al., 2021). We performed similar analyses on our dataset to look at the activity of M1 and CB units during spindles identified in the M1 and CB. To observe spindle–neuron interactions, we aligned spike rasters of M1 and CB units to the peak of identified, respectively determined local spindles. The average oscillatory firing rate of M1 neurons closely matched (with a phase shift) the average spindle waveform of M1 spindles and CB spindles (Fig. 3*A* shows an example M1 unit spiking around M1 spindles; Fig. 3*B* shows the same M1 unit spiking around CB spindles). CB units’ firing also increased around the peak of CB and M1 spindles and followed the oscillations of the CB/M1 spindle waveform in a subset of neurons (Fig. 3*C* shows an example CB unit spiking around M1 spindles; Fig. 3*D* shows the same CB unit whose firing rate tracked the waveform of CB spindles without a phase shift).

**Figure 4.**
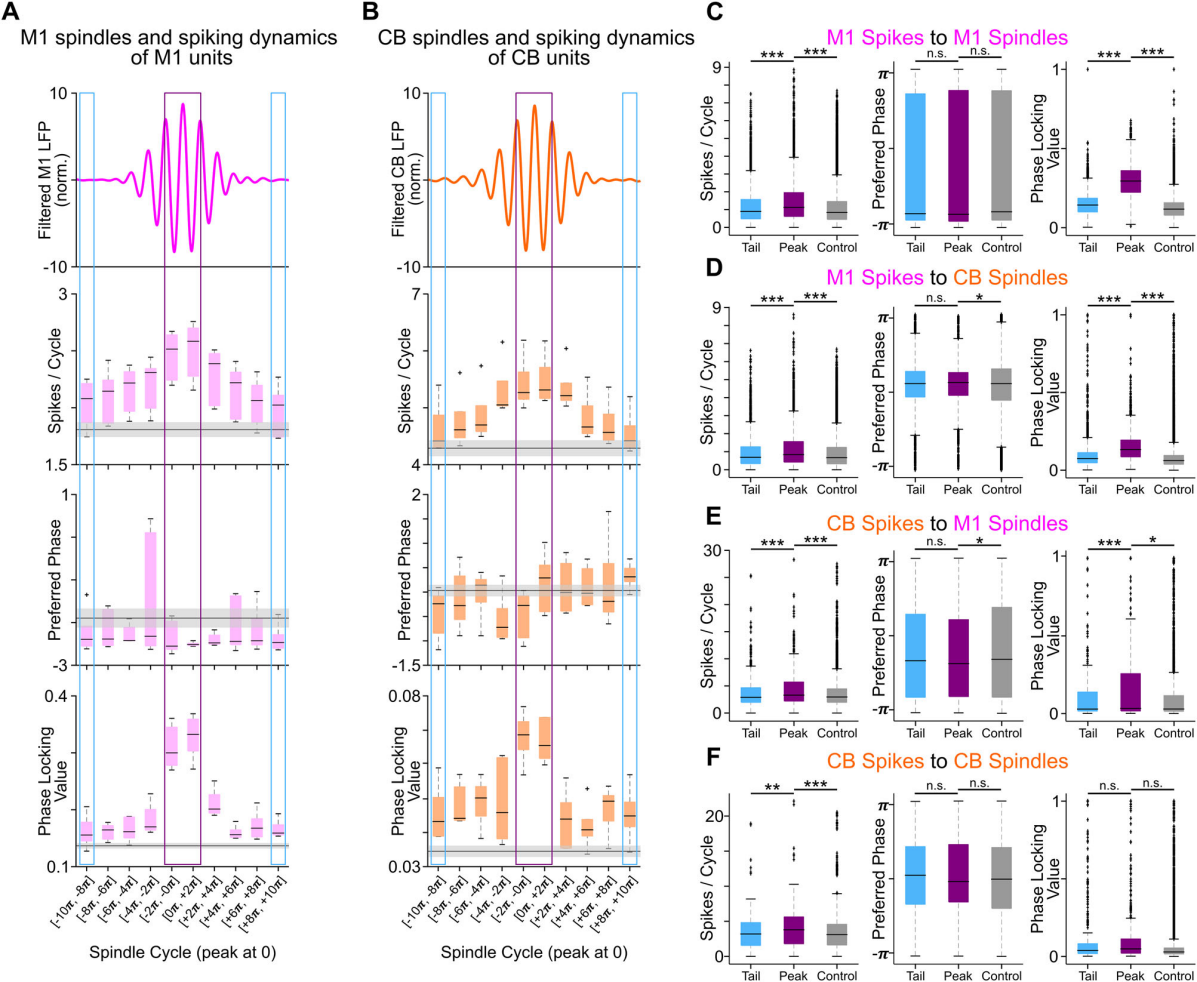
Spindle cycle analysis of M1 and CB spiking. ***A***, Summary of an example animal's all M1 spike phase distribution statistics across M1 spindle cycles. The average spindle band waveform for each spindle cycle (top) is plotted along with the average spike rate (second from top), preferred phase angle (second from bottom), and PLV (bottom). Lower and upper box boundaries are 25th and 75th percentiles, respectively; line inside the box is the median; lower and upper error lines are 10th and 90th percentiles, respectively; “+” indicates outliers outside these bounds. ***B***, Same as ***A*** but for CB spiking activity across CB spindles in an example animal. Conventions are the same. ***C***, Summary of M1 spiking dynamics related to M1 spindles: spike rate (left), preferred phases (middle), and PLVs (right) are combined into three categories: (1) tail, the two cycles furthest from spindle peaks; (2) peak, the two cycles nearest the spindle peaks; and (3) control, the two cycles at the center of control epochs from expert animals (*n* = 4). ***D***, Same as ***C*** but it depicts summary statistics for M1 spiking activity during CB spindle cycles ***E*** and ***F***, Summary of spiking rate dynamics as in ***C*** and ***D*** but for CB spikes around M1 spindles and CB spindles, respectively. *p < 0.05. ***p < 0.001. n.s.: not significant.

To quantify this, we extracted the spindle phase at each recorded action potential. To compute the spindle phase, we calculated the angle of the Hilbert-transformed, spindle band LFP. Then we collected the phase triggered on each spike occurring within one cycle of the spindle peak. This yielded a spike phase distribution (Fig. 3*A–D*, bottom), which was used to calculate the degree of phase locking for each single unit. Briefly, each spike-triggered phase was converted to a vector of unit magnitude and in the direction of the triggered phase. Then the average vector was computed, and the magnitude of this vector was taken as the PLV, while the direction of this vector was taken as the preferred spindle phase. The polar histograms in Figure 3 depict preferred phases and PLVs for three units in the two cycles around the spindle peak ([−2π, +2π]) and two cycles on the either spindle tail (i.e., [−10π, −8π] and [+8π, +10π]). These are shown in a green line on top of the polar histogram (Fig. 3*A–D*, bottom). When we compared the PLV of all the neurons aligned to their respective spindles, we found that M1 spiking aligned to M1 spindles shows a greater increase in PLV around the spindle peak than the tail (Fig. 3*E*) and M1 spiking aligned to CB spindles (Fig. 3*F*). Similarly at the population level, cerebellar spiking aligned to M1 spindles shows an increase in PLV at the peak versus the tail (Fig. 3*G*); however, cerebellar spiking aligned to CB spindles showed a nonsignificant change in PLV at the peak versus the tail (see Fig. 3*H* and more details in Fig. 4*F*). The stronger phase locking of M1 and CB spiking during M1 spindles suggests that M1 spindles are more robustly modulating spiking activity of both these regions than CB spindles.

### Single-unit phase locking around M1 and CB spindles

In addition to increasing spiking, phase locking to ongoing oscillations is also linked to long-term potentiation (Rutishauser et al., 2010). Our next analysis focused on the detailed phase-locking analysis of several cycles within a spindle for both M1 and CB spindles, as was recently done for M1 spiking and M1 spindles (Silversmith et al., 2020). Calculating meaningful population means and significance tests of the preferred phase and PLVs requires the neural population to have a nonuniform distribution of the preferred phases. The population of M1 units had nonuniform phase distribution with respect to M1 and CB spindles. CB unit population had nonuniform preferences with respect to M1 spindles but uniform preferences with CB spindles (Fig. 4).

To analyze unit modulation at various cycles within a spindle, a spindle was segmented into 10 cycles (Fig. 4*A,B*). We also looked at same unit's modulation during control epochs, which were also divided into component cycles and yielded a spike phase distribution for each cycle, as done in recent work (Silversmith et al., 2020; also see Materials and Methods). The spiking dynamics for M1 and CB units were quantified by grouping LFP cycles into three categories: (1) control, the two cycles at the center of the control epochs; (2) tail, the two cycles farthest from the spindle peaks; and (3) peak, the two cycles nearest the spindle peaks (Fig. 4*C–F*). Linear mixed-effects model confirmed that spike counts were significantly increased near the peak of spindles [peak vs tail, M1 spikes to M1 spindles, *t*_(5,086)_ = 8.68, *p* = 5.251 × 10^−18^; M1 spikes to CB spindles, *t*_(5,086)_ = 8.63, *p* = 7.173 × 10^−18^; CB spikes to M1 spindles, *t*_(910)_ = 3.40, *p* = 6.752 × 10^−4^; CB spikes to CB spindles, *t*_(910)_ = 2.89, *p* = 0.003]; spike counts were also significantly higher at the spindle peaks relative to during control epochs [peak vs control, M1 spikes to M1 spindles, *t*_(7,982)_ = −16.86, *p* = 1.577 × 10^−63^; M1 spikes to CB spindles, *t*_(7,982)_ = −5.17, *p* = 2.297 × 10^−7^; CB spikes to M1 spindles, *t*_(5,014)_ = −15.49, *p* = 6.012 × 10^−54^; CB spikes to CB spindles, *t*_(5,014)_ = −4.96, *p* = 7.099 × 10^−7^]. M1 units’ preferred M1 and CB spindle cycle phases (as determined by the Watson–Williams test for equal means) did not change between peak, tail, and control epochs (although one test showed a significant difference in the preferred phase between peak vs control for M1 spikes and CB spindles and CB spikes and M1 spindles; this phase difference was <0.01*π*; Fig. 4*D,E*). Like spike count, phase locking also increased near spindle peaks except for CB spikes aligned to CB spindles [peak vs tail, M1 spikes to M1 spindles, *t*_(5,086)_ = 8.68, *p* = 0; M1 spikes to CB spindles, *t*_(5,086)_ = 55.29 *p* = 0; CB spikes to M1 spindles, *t*_(910)_ = 4.72 *p* = 2.488 × 10^−6^; CB spikes to CB spindles, *t*_(910)_ = 0.64, *p* = 0.52]. PLV was also significantly larger around spindle peak than control epochs even five cycles away for every pair except for CB spikes aligned to CB spindles [peak vs control, M1 spikes to M1 spindles, *t*_(7,982)_ = −106.89, *p* = 0; M1 spikes to CB spindles, *t*_(7,982)_ = −8.19, *p* = 2.971 × 10^−16^; CB spikes to M1 spindles, *t*_(5,014)_ = −2.07, *p* = 0.03; CB spikes to CB spindles, *t*_(5,014)_ = −1.37, *p* = 0.16].

### Percentage of M1 and CB units with nonuniform spike phase distributions around spindles in expert versus nonexpert learners

**Figure 5.**
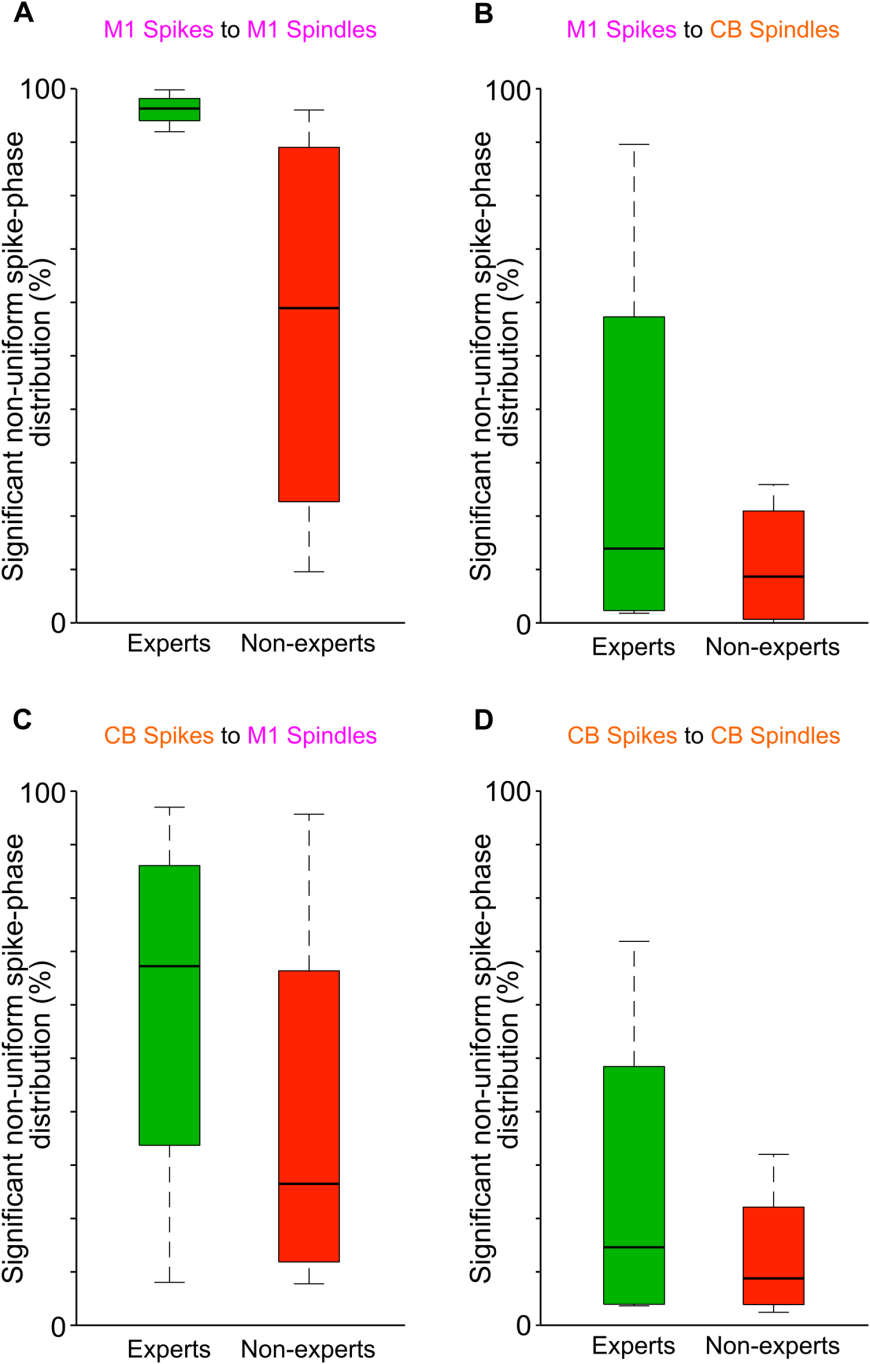
Significantly nonuniform spike phase distribution around M1/CB spindle peaks. ***A***, Boxplot showing percentage of M1 unit–M1 spindle pair with significant *z*-statistics from Rayleigh's test for circular nonuniformity using M1 spikes at the M1 spindle peak, in expert (*n* = 4) and nonexpert learners (*n* = 4). The significance threshold of the *z*-statistic was set at *p* < 0.05. Lower and upper box boundaries are 25th and 75th percentiles, respectively; black line inside the box is the median; lower and upper error lines are 10th and 90th percentiles, respectively. ***B***, Same as ***A*** but for M1 unit–CB spindle pairs; conventions are the same. ***C***, Same as ***A*** but for CB unit–M1 spindle pairs; conventions are the same. ***D***, Same as ***A*** but for CB unit–CB spindle pairs; conventions are the same.

PLV is directly related to the *z-*statistic of the Raleigh's test for circular nonuniformity with *z* = *n*(PLV)^2^. We took a closer look at the ratio of neurons with a significant phase preference (i.e., *p*-value of the *z-*statistic <0.05). We found a higher number of units with nonuniform spike phase distributions at the spindle peaks in expert learners versus nonexpert learners [Fig. 5; M1 spikes to M1 spindles, peak experts, 96.1%; peak nonexperts, 55.8% (% indicates the percentage of units with significant nonuniform spike phase distribution around spindle peaks); M1 spikes to CB spindles, peak experts: 29.8%, peak nonexperts: 10.8%; CB spikes to M1 spindles, peak experts: 59.8%, peak nonexperts: 39.1%; CB spikes to CB spindles, peak experts: 26.2%, peak nonexperts: 13.0%].

### Distribution of PLV between expert and nonexpert learners

**Figure 6.**
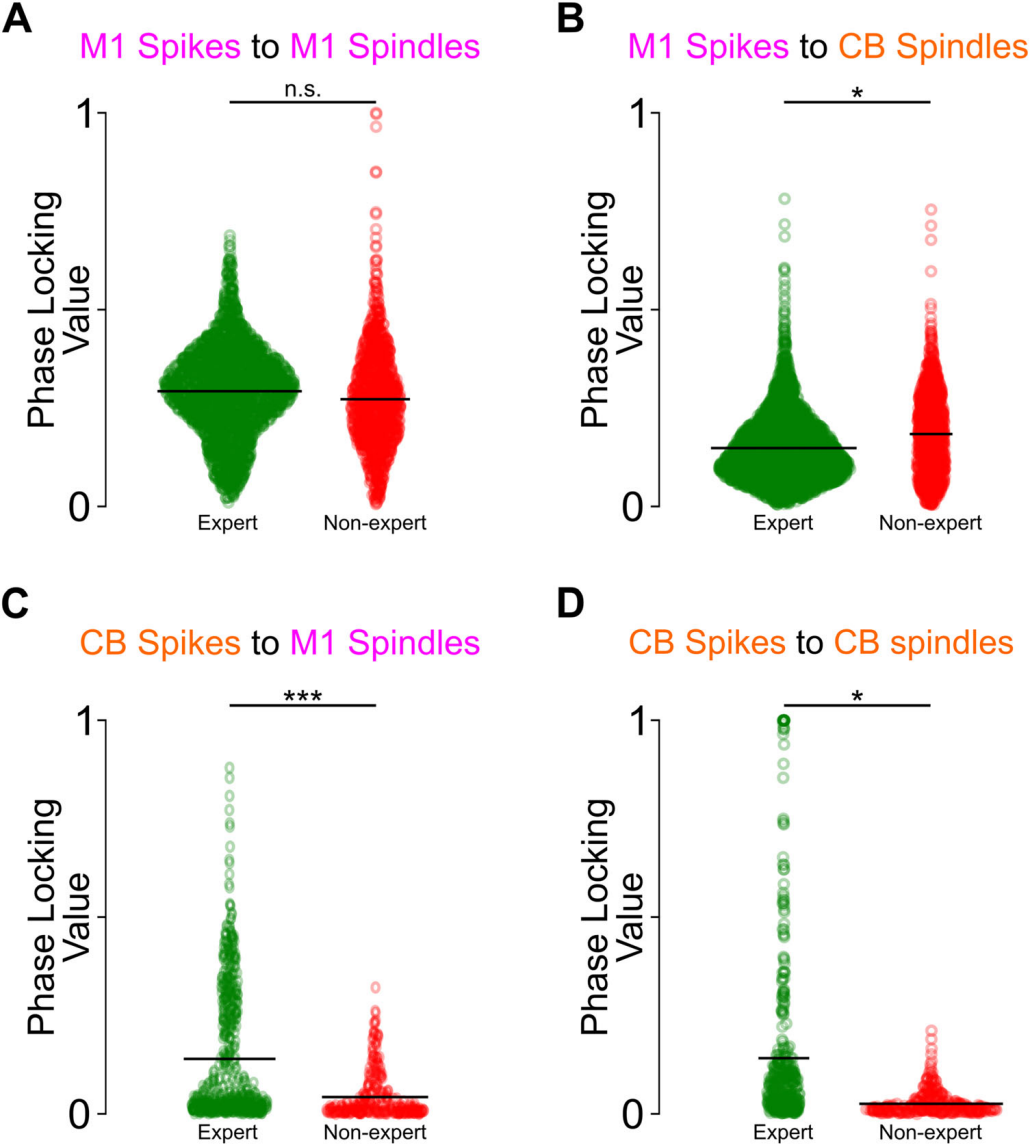
Distribution of PLV during spindle peaks in expert versus nonexpert learners. ***A***, PLVs for expert (green; *n* = 4) and nonexpert (red; *n* = 4) learners. Each dot represents PLV of a single M1 unit–M1 spindle peak pair. Black vertical line represents the mean. ***B***, Same as ***A*** but for M1 unit–CB spindle peak pairs; conventions are the same. ***C***, Same as ***A*** but for CB unit–M1 spindle peak pairs; conventions are the same. ***D***, Same as ***A*** but for CB unit–CB spindle peak pairs; conventions are the same. *p < 0.05. ***p < 0.001. n.s.: not significant.

Next, we wanted to compare the PLV in the expert versus nonexpert animals. We found higher PLV in expert when compared with nonexpert learners across all pairs except for M1 spikes and CB spindles [Fig. 6; M1 spikes to M1 spindles: expert learners 0.29 ± 0.002, nonexpert learners 0.27 ± 0.003, *t*_(3,719)_ = −1.43, *p* = 0.15; M1 spikes to CB spindles: expert learners 0.14 ± 0.001, nonexpert learners 0.18 ± 0.003, *t*_(4,398)_ = 2.08, *p* = 0.03; CB spikes to M1 spindles: expert learners 0.13 ± 0.007, nonexpert learners 0.04 ± 0.003, *t*_(973)_ = −8.83, *p* = 4.661 × 10^−18^; CB spikes to CB spindles: expert learners 0.14 ± 0.01, nonexpert learners 0.02 ± 0.001, *t*_(745)_ = −2.31, *p* = 0.02]. Thus, interestingly, while we observed that a higher number of expert learners’ units had significantly nonuniform spike phase distribution for M1 unit–CB spindle (Fig. 5*B*), their averaged PLV was less than that of nonexpert learners (Fig. 6*B*).

### Increase in spindle band LFP coherence is correlated with motor learning

**Figure 7.**
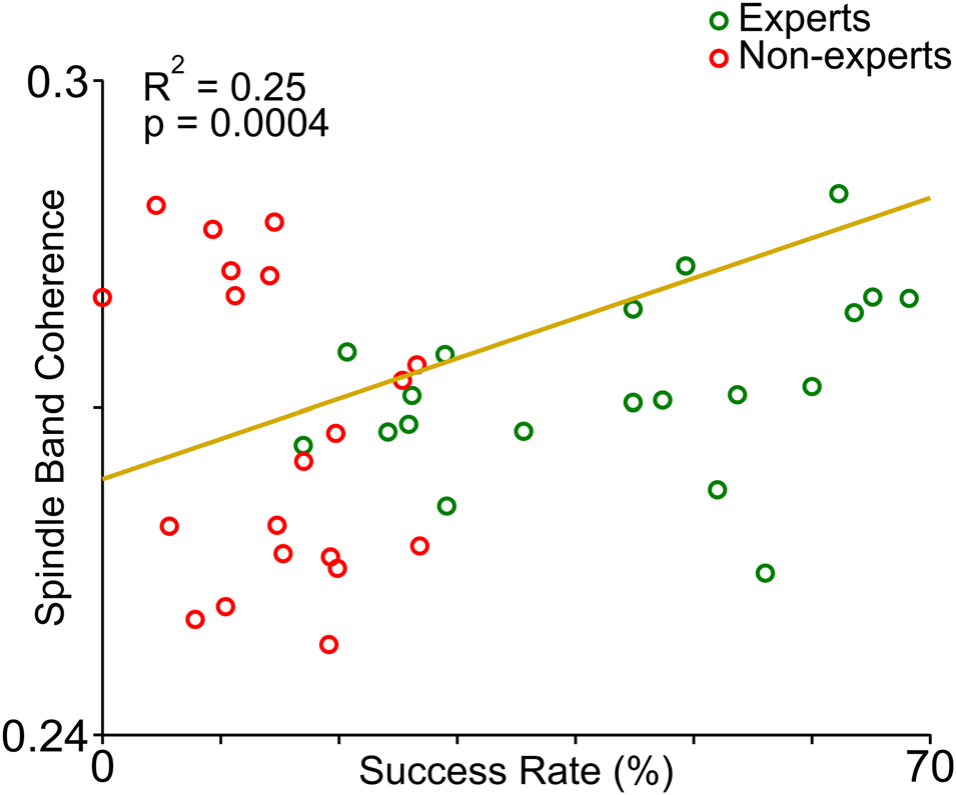
Relationship between M1 and CB spindle band LFP coherence during sleep and awake training success rate. Correlation between spindle band coherence and success rate in expert (*n* = 4) and nonexpert (*n* = 4) learners.

Our recent work with the same animals showed that awake low-frequency coherent activity (LFOs, 1–4 Hz) in M1 and CB LFPs did not increase in nonexpert animals with learning (Fleischer et al., 2023). We wanted to see if spindle band coherence in M1–CB LFPs during sleep was linked to eventual skill consolidation or “expertise” level. Hence, we looked at the relationship between M1 and CB spindle band (10–16 Hz) LFP coherence magnitude with learning. Interestingly, we found that high 10–16 Hz coherence in M1–CB LFPs during sleep showed a positive correlation with success rate in awake training (Fig. 7).

## Discussion

In this study, we investigated the relationship between M1 and CB neural firing and LFP spindle oscillations during sleep. We focused on the structure of neural spiking during the component cycles of both M1 and CB spindles (i.e., peak cycles and tail cycles). Spindles are thought to be important for promoting neural plasticity after learning a new skill (Fogel and Smith, 2006; Nishida and Walker, 2007; Barakat et al., 2011; Johnson et al., 2012; Ramanathan et al., 2015; Kim et al., 2019; Silversmith et al., 2020; Lemke et al., 2021). We found that M1 units fired at a preferred phase of M1 and CB spindles and CB units fired to a preferred phase of M1 spindle but not the CB spindles (Figs. 3, 4). Both M1 and CB neurons, however, did have elevated spike rate at M1/CB spindle peaks. Notably, the animals that gained expertise in a skilled reaching task had higher numbers of neurons in the M1 and CB that developed a nonuniform spike phase distribution to M1 or CB spindle peaks (Fig. 5). Finally, we found that spindle band LFP coherence in M1 and CB LFPs during sleep was positively correlated to the awake reach-to-grasp task success rate (Fig. 7). These findings support the idea that sleep spindles in both corticocerebellar networks modulate neurons that participate in the awake task to support motor memory consolidation.

### Spindle modulation of spiking and plasticity

One of the key findings from our work is that there were selective changes in the degree of phase locking of M1 spiking across spindle cycle peaks of M1 and CB spindles and CB spiking across M1 spindle peaks (Fig. 4). While we found that spike timing in the M1 became significantly more coupled to the structure of M1 and CB spindles, leading to maximum changes at the spindle peaks (Fig. 4*C,D*, right), CB spiking did not develop a phase preference relative to CB spindle peaks, but rather to M1 spindle peaks (Fig. 4*E,F*, right) in the rats that learned reach-to-grasp motor task expertly. This was surprising as we saw greater spikes per cycle for CB units at CB spindle peak cycles (Fig. 4*F*, left). Previous work has shown that neocortical neurons also display increased correlated discharge around neocortical spindle peaks (Silversmith et al., 2020). Additionally, there is evidence that sleep spindles mediate corticostriatal coupling with increased correlated spiking of M1 and striatal neurons (Lemke et al., 2021), and such spike correlations are thought to drive neuroplasticity (Hebb, 1949). Spike time-dependent plasticity models (Bi and Poo, 1998; Shulz and Jacob, 2010; Feldman, 2012) emphasize the role of precise spike timing in neuroplasticity. While the experimental studies cited above showed increased correlated spiking during a spindle within local pairs of M1 neurons or monosynaptically connected M1–striatal neurons, we report increased spike rates of M1 and CB neurons during local or cross-area spindle peaks (Fig. 4*C–F*). Notably, these neurons were also modulated during awake reach-to-grasp task training. The areas we recorded from (i.e., M1 layer IV/V) and the cerebellar cortex (Simplex, Crus I, and Crus II) are not connected monosynaptically but primarily through the corticopontocerebellar pathway (Guo et al., 2021). It may be possible that the increased spike rates of M1 and CB neurons observed at spindle peaks serve to strengthen their monosynaptic connections within this pathway. This may facilitate a general increase in local functional connectivity which subserves the reaching skill.

It is important to note that in this work, we measured the modulation of M1 and CB units during local and cross-area spindle cycles. One possibility is that the increased firing at spindle peaks (Figs. 3, 4) reflect changes in the synaptic strength of neurons that are a part of the corticopontocerebellar pathway. We previously found increased awake low-frequency LFP coherence between the M1 and CB (LFOs, 1–4 Hz) that also modulated M1 and cerebellar spiking in rats that gained expertise in the reach-to-grasp task (Fleischer et al., 2023). This likely indicated that cortical inputs to and/or from the CB were strengthened with motor training, which is consistent with other works that have looked at emergent activity in corticocerebellar networks with motor training (Wagner et al., 2019; Wagner and Luo, 2020). An alternative possibility is that inputs to both the M1 and CB during spindles drove these spike modulations. Our comparison on M1 and CB spindle onset lags shows that there were spindles that occurred in close temporal proximity in these regions (Fig. 2*C*), and recent studies have shown that neocortical spindles have a cerebellar origin (Xu et al., 2021). We believe that our results are most consistent with increased synaptic strength in M1 to CB connectivity during NREM, as we observed increased LFP coherence during awake task performance in these same rats (the ones that learned the task well).

### Skill learning in corticocerebellar networks

Here we show that the spiking activity of M1 and cerebellar neurons is heightened during spindle peaks in either region and that there are a higher number of M1 and CB neurons with nonuniform spike–LFP phase distribution to spindle peaks in rats that achieved expertise in skilled reaching. We make these observations, building on our recent dataset where we found increased coordination of awake M1 and cerebellar activity with skill acquisition (Fleischer et al., 2023). Increased communication in the M1 and CB has been noticed in other works as well which is essential to stable skilled behavior (Wagner et al., 2019; Guo et al., 2021). We have found that nonexpert animals do not develop awake M1–CB coordination, that is, the low-frequency (1–4 Hz) coherence (Fleischer et al., 2023), and these animals also show reduced number of neurons with nonuniform spike phase distribution to M1/CB spindle peak cycles (Fig. 5). These observations are consistent with a range of other studies that show that coordinated sleep activity benefits consistency in motor tasks in humans (Fischer et al., 2002; Walker et al., 2002) and rodents (Gulati et al., 2014; Ramanathan et al., 2015; Nagai et al., 2017).

While the directionality in our M1–CB recordings may be preconceived to be predominantly from M1 to CB via the pons (Kelly and Strick, 2003), they are heavily reciprocally connected (Heck et al., 2013). While evidence exists for coordination in these regions with skill learning (Wagner et al., 2019; Fleischer et al., 2023), this directionality comes into conflict with recent studies postulating sleep spindles to have a cerebellar origin (Xu et al., 2021). Our analysis of lags shows that some CB spindle onset precedes M1 spindle onset, but there are CB spindles that followed M1 spindles (Fig. 2*C*). It is important to note that in the M1–CB reciprocal loop, deep nuclei in the CB are the major outflow node from the CB back to the cortex via the motor thalamus. Studies have shown that cerebellar deep nuclei activity can control forelimb deceleration during reaching task (Becker and Person, 2019) and that thalamic input is essential for reliable cortical neural dynamics (Sauerbrei et al., 2020). Hence, within the CB resides a cause of M1 activation as well as its consequence. Interestingly, in our awake recordings, we found that cerebellar neurons phase-locked more with M1 emergent low-LFO activity (Fleischer et al., 2023), and this trend was preserved during sleep, where CB neurons showed enhanced phase locking to M1 spindle peaks (and not to CB spindle peaks) in expert rats (Fig. 4*E,F*). It is possible that during sleep spindles, the communication from the M1 to CB is preserved, and it is also possible that this direction of communication evolves with learning, wherein during initial phases, cortical input to the CB is important and with well-learned, stable movement, cerebellar feedback to M1 becomes critical. Future work will be needed to determine if sleep facilitates changes in the direction of communication between the M1 and CB.

In conclusion, our results demonstrate that neural activity in the M1 and CB is modulated during local and cross-area spindles very precisely at spindle peaks with M1 activity also developing a preferred phase to each area's spindle peak cycle and CB activity developing this preference only to M1 spindle peak. Furthermore, we found a higher number of neurons that developed a nonuniform spike phase distribution to spindle peaks in animals that gain expertise in the skilled motor task. These findings help build a framework to study the relationship between changes in precisely structured spiking activity during local and cross-region spindles. Our work also suggests an off-line neural processing mechanism that may drive synaptic plasticity associated with motor learning in the larger corticocerebellar network.

## Synthesis

Reviewing Editor: Michaël Zugaro, CNRS, Collège de France, Inserm Decisions are customarily a result of the Reviewing Editor and the peer reviewers coming together and discussing their recommendations until a consensus is reached. When revisions are invited, a fact-based synthesis statement explaining their decision and outlining what is needed to prepare a revision will be listed below. The following reviewer(s) agreed to reveal their identity: Clément Léna.

The revised manuscript has been substantially improved. Some revisions and language editing are still required to complete the review process.

- Title: the authors may wish to specify the motor cortex-cerebellar network.

- In many instances in the text, the authors refer to spindles as 'active' modulators of spiking. This could be rephrased as a more descriptive language, e.g. on line 37 the 'effect of spindles' could be more appropriately described as spindle-associated spiking activity. Similarly, on line 78, 'reactivated by spindles' should be reformulated as 'reactivated during spindles'. Other examples include line 108 'spiking affected by spindles' (change to e.g. 'spiking during spindles'), lines 315 and 348 'spindle modulation of spiking' (change to 'spindle-associated spiking modulated by motor skill learning'), line 350 spindles 'increased' phase-locking, etc. Overall, spindles are often metaphorically depicted as 'acting' on neurons, which may be misleading for a non-expert reader.

- A higher firing rate and phase-locking at the spindle peak are expected as the amplitude of spindle oscillation indicates the entrainment of a larger neuronal population, at least locally.

- The authors do not find temporal relationships between the Cb neurons and Cb spindles. How heterogeneous were Cb units? Could Cb unit heterogeneity account for the lack of spike-spindle coupling? It is not clear what neural elements contribute to the Cb spindle generation.

- Both in M1 and Cb spindles are rather short (~1s). The spindle average frequency is at the very lower border of the conventional spindle range. Was it a subclass of low-frequency spindles or the range 10-16Hz was not optimal? Was there a distinct increase in the spindle power on the power spectrum? Finally, do M1 and Cb spindles co-occur with spindles detected in the EEG? Do they reflect local or more global neuronal synchronization?

- How was the spindle tail defined? Was it bi-sided (data combined for both tails)?

- Information on the single unit extraction and unit basic features (spike width, firing rate, etc.) is still missing.

- Experts and non-experts. It is still not clear if the data presented in Figs.2,3, and 4 come from 4 and 8 animals. If only experts were used, it is unclear why non-experts were excluded from these analyses.

Typographical errors

- Line 53: rephrase 'sleeping rats' to e.g. 'naturally behaving rats' or 'during spontaneous activity'...

- Line 57: 'M1/cerebellum spindles' a slash may be interpreted as temporally coupled events.

- Line 59: 'except for' the lack of coupling is important and should be described separately.

- Line 74: 'awake practice neuronal (?) activity' please clarify

- Line 85: revise citations, not all cited studies used a declarative memory task.

- Line 99: revise 'cross-area' spindle-mediated interactions (?).

- Line 111: explain 'cerebellar involvement' more clearly.

- Line 118: 'increased' compared to what?

- Line 121: does 'a greater number' actually mean a significantly more neurons? please clarify.

- Line 298: 'one sample' please clarify

- Line 346: does 'modest' mean non-significant?
